# Non-suicidal self-injury and its risk factors among Iranian high school and university students

**DOI:** 10.3389/fpsyt.2024.1425411

**Published:** 2024-10-17

**Authors:** Abbas Abbasi-Ghahramanloo, Behnam Khodadoust, Davoud Adham, Ali Gholami, Roya Farokhi, Vahid Maghsodlou-Nejad, Sima Afrashteh

**Affiliations:** ^1^ Department of Public Health, School of Health, Ardabil University of Medical Sciences, Ardabil, Iran; ^2^ Department of Epidemiology, School of Health, Iran University of Medical Sciences, Tehran, Iran; ^3^ Non communicable Diseases Research Centre, Neyshabur University of Medical Sciences, Neyshabur, Iran; ^4^ Department of Epidemiology & Biostatistics, School of Public Health, Neyshabur University of Medical Sciences, Neyshabur, Iran; ^5^ Department of Epidemiology & Biostatistics, School of Public Health, Ilam University of Medical Sciences, Ilam, Iran; ^6^ Department of Epidemiology, Shahroud University of Medical Sciences, Shahroud, Iran; ^7^ Department of Biostatistics and Epidemiology, Faculty of Health and Nutrition, Bushehr University of Medical Sciences, Bushehr, Iran

**Keywords:** non-suicidal self-injury, high school students, university students, prevalence, Iran

## Abstract

**Background:**

Non-suicidal self-injury (NSSI) is an important health problem among adolescents and young adults. The aims of this study were to determine NNSI status and its associated factors among students.

**Method:**

This cross-sectional study has been conducted in 4715 high school and university students from the West and Northwest provinces of Iran. Multi-stage sampling was used to select students and all students completed survey questionnaires. Data analysis was performed using chi-square, Fisher’s exact test, binary and logistic regression.

**Result:**

The lifetime prevalence of NSSI was estimated as 24.4% among all participants (21.4% in high school students vs. 29.3% in university students). After adjustment for other factors, age (OR=1.08,95%CI:1.03-1.12), cigarette smoking as an experimenter (OR=1.94,95%CI:1.61-2.34) and regular (OR=3.80,95%CI:2.96-4.89) compared to nonsmokers, membership in a sport team (OR = 1.26,95%CI:1.08-1.48), having physical fight (OR = 2.18,95%CI:1.85-2.57), doing general risk taking behavior (OR = 2.05,95%CI:1.66-2.53), and self-esteem (OR=0.93,95%CI:0.92-0.95) were associated with experience of NSSI.

**Conclusion:**

Our results suggested that the prevalence of NSSI was relatively high among high school and university students. To diagnose, prevent, and treat NSSI in teenagers and young adults in Iran, more research is needed to identify the root causes.

## Introduction

Non-suicidal self-injury (NSSI) (is an important health problem among adolescents and young adults (1). This behavior is usually occurring in the form of cutting, burning, scratching, and hitting the body and often in order to escape from sadness and anger and cognitive states (2).

The prevalence of NSSI varies in different studies due to varying definitions of NSSI (3, 4). The results of a review study in six geographical regions (Asia, Australia/New Zealand, Canada, Europe, United Kingdom, USA) over19 years, reported the prevalence of NSSI ranging from 1.5 to 54.8% (5). The high prevalence of NSSI has been reported in the western countries from 17.2% in American to 20.1% in European adolescents and in the high school students were 20% and 10.3% for American and European, respectively (5, 6). This behavior is also prevalent in Asian countries; the results of studies among students of Japan, India, and Turkey have reported a prevalence of NSSI of 10, 31.2, and 28.5%, respectively (7, 8). In addition, in a study among Chinese high school students, Siying et al. showed that the prevalence of NSSI was 28.9%, with girls suffering more than boys (9). The prevalence of NSSI among adolescents and young people in Iran has been reported as 39.3% and 12.3%, respectively (10, 11).

The results of various studies indicated that NSSI is associated with a variety of mental illnesses, smoking, alcohol and drug use, poor family functioning, age gender, and previous year average grade (4, 12, 13). This behavior can lead to serious physical consequences, infectious diseases, medical complications, emotional distress, social isolation, academic dysfunction, and eventually death (14). Experiencing NSSI reduces the fear and pain of death and thus increases the ability to attempting suicide. Therefore, this behaviors are an important predictor for future attempted or completed suicide (2, 15).

Evidence shows that self-harm behavior can be caused by various factors, such as biological, physiological, genetic, psychological, social, and cultural factors (16, 17). Specifically, NSSI has been found to be more prevalent in patients with bipolar disorders, polar disorders, anxiety disorders, and substance abuse, in particular (16). Negative life events, adverse childhood experiences, having weak family relationships, having harsh or controlling parents, and experiencing discrimination can be long-term environmental and social risk factors for NSSI. So that abuse during life, life problems and family dysfunction makes a person susceptible to injury and NSSI with the aim of modulate affective or social dysregulation (16, 18). However, high-level social support and self-esteem have been identified as protective factors for NSSI. People with high self-esteem feel valued and in contrast people with low self-esteem and low-level social support are prone to self-destruction, feelings of shame and guilt, excessive irritability and anger, having negative feelings about yourself, a pessimistic view of life, and ultimately NSSI (19, 20). According to Iranian studies, NSSI is caused by factors such as mental disorders, depression, self-concept disturbance, and weak family psychological function (12, 21).

Most self-injurious behaviors resolve over time without treatment. However, sometimes NSSI serve as a gateway to suicide (22, 23). Understanding the risk factors of NSSI in developing countries including Iran with large portion of adolescents and young adults is of great importance. Therefore, the present study was conducted to determine NSSI status and its determinants among a large sample of high school and university students in Iran.

## Methods

### Participants and procedures

This cross-sectional study was performed on 4715 high school and university students. University students were selected from four universities in Ilam in western Iran from March to June 2013. Multi-stage sampling was used to select students and the type of university was considered a stratum, and the number of classes was considered a cluster. Finally, 2000 students were invited to participate in the study.

High school students were also selected from the cities of Zanjan (northwest of Iran) and Ilam (West of Iran) from July 2013 to April 2014. In Zanjan, students were randomly selected from 61 classrooms in 42 high schools. At the beginning of the study, there were 12,000 high school students in Zanjan, out of which a total of 1,100 students were selected using multi-stage sampling. In Ilam, with a similar approach, 120 classes were selected from 75 high schools, and finally 2000 students entered to the study. The total number of high school students enrolled in the study was 3,100.

Before collecting the data, explanations were given to the students in each class by trained interviewers. These explanations were about the study’s objectives, the anonymity of the questionnaires and the informed consent form and how to complete the questionnaire. The ethics committee of Ilam and Zanjan University of Medical Sciences approved this study and all study participants signed an informed consent form. Further details on the study have been published in other articles (24, 25).

### Measures

A valid and reliable questionnaire previously made in Iran was used to collect data on high-risk behaviors (26).This questionnaire is designed for Iranian students and includes information on smoking and hookah, alcohol and drugs. Other variables used in this study include physical fight, membership in sport teams, socioeconomic status and self-esteem. General risk taking behavior was measured using the Kaplan method (27). NSSI was assessed using a direct question. Students were asked whether they had a history of NSSI if the answer was yes, they were asked to declare the type of NSSI. Physical fight also assessed using the direct question, “Have you had a physical fight with anyone in the past year?” Self-esteem was measured using the Persian version of the Rosenberg scale. The purpose of this tool is to evaluate how a person assesses their worth as a human being. It has good psychometric properties (Cronbach’s alpha coefficient = 0.88) in Iran. The items of the questionnaire were evaluated on a Likert scale from 1 to 4, and the answers were rated as “completely agree = 4”, “agree = 3”, “disagree = 2” and “completely disagree = 1”.The range of scores spanned from 10 to 40, with a higher score indicating a higher level of self-esteem (28). Socioeconomic status was constructed using the father’s education, mother’s education, family assets, and income using PCA. Finally, the study participants were divided into three categories (low, middle, and high) based on socioeconomic status. The study was approved by the Ethics Committee of Ilam University of Medical Sciences (917013/132). All students had signed an informed consent form. More details of this study have been reported elsewhere.

### Statistical analysis

For categorical and quantitative variables, frequencies (%) and mean (SD) are reported respectively. Chi-square and Fisher’s exact tests were used to analyze the data and examine the relationship between each of the qualitative variables and NSSI. The independent samples t-test was used to compare the mean scores of quantitative variables between participants who had NSSI and those who did not. Univariate and multivariate logistic regression analysis was used to estimate the crude and adjusted odds ratios, respectively. The Hosmer-Lemeshow strategy was used to select the variables in multiple logistic regression (29). Analyzes were performed using SPSS 16 software and P-value less than 0.05 was considered as a significant level.

## Results

### Demographic characteristic

Out of 5100 participants in the study, 4715 people had completed the questionnaires in a usable way (Response rate = 92.4%). The mean age of study participants was 19.10 ± 3.24 years. About 62% of the study participants were high school students. The majority of participants (80.4%) were male. Among all participants of this study, 1166 (24.4%) reported NSSI experience. **Table 1** shows the relationship between demographic characteristics and some factors affecting NSSI. As can be seen in **Table 1**, being male, cigarette smoking, membership in sport teams, physical fight, general risk-taking behavior, living with parents and age showed a significant relationship with NSSI (P<0.05). While the socioeconomic status and the number of close friends were not significantly associated with NSSI (P>0.05).

**Table 1:** Demographic characteristics and risk taking behaviors by non-suicidal self-injury in a sample of Iranian students.

Characteristics	Non-suicidal self-injury		Total	
	No	Yes		p-value
Gender, n (%)				
Male	2792 (73.7)	996 (26.3)	3788	<0.001
Female	757 (81.7)	170 (18.3)	927	
Student of, n (%)				
High School	2273 (78.2)	632 (21.8)	2905	<0.001
University	1276 (70.5)	534 (29.5)	1810	
Cigarette smoking status, n (%)				
Non smoker	2764 (82.8)	575 (17.2)	3339	<0.001
Experimenter	566 (62.8)	335 (37.2)	901	
Regular smoker	184 (43.4)	240 (56.6)	424	
Number of close friends, n (%)				
None	65 (73.0)	24 (27.0)	89	0.620
1-5	2090 (74.9)	700 (25.1)	2790	
More than 5	799 (76.2)	249 (23.8)	1048	
Membership in a sport team, n (%)				
No	2041 (77.4)	596 (22.6)	2637	<0.001
Yes	1428 (72.4)	544 (27.6)	1972	
Physical fight, n (%)				
No	2358 (82.3)	508 (17.7)	2866	<0.001
Yes	1148 (64.2)	639 (35.8)	1787	
General risk taking behavior, n (%)				
No	1079 (86.9)	163 (13.1)	1242	<0.001
Yes	2459 (71.2)	993 (28.8)	3452	
Living with parents, n (%)				
No	159 (67.4)	77 (32.6)	236	0.003
Yes	3252 (75.9)	1034 (24.1)	4286	
Socioeconomic status, n (%)				
Low class	788 (75.0)	263 (25.0)	1051	0.318
Middle class	846 (77.1)	251 (22.9)	1097	
High class	851 (77.1)	246 (22.9)	1097	
**Age, mean (SD)**	18.86 (3.15)	19.85 (3.44)	19.10 (3.24)	<0.001
**Self-esteem, mean (SD)**	31.65 (5.36)	29.02 (5.82)	30.99 (5.58)	<0.001

### Factors associated with NSSI

A logistic regression model was used to estimate the crude ORs for all of the variables (**Table 2**). The results of this analysis indicated that, all variables have a significant effect on NSSI. Among these variables, regular cigarette smoking had the highest OR and only self-esteem was a protective factor for experience of NSSI.

**Table 2:** Univariate logistic regression analysis of the association between “non-suicidal self-injury" and predictors in a sample of Iranian students.

	Non-suicidal self-injury		
	*Crude estimation*		
Variables	OR	95%CI	P
**Gender (being male)**	1.59	1.32-1.90	<0.001
**Being university student**	1.50	1.32-1.72	<0.001
Cigarette smoking status			
Non smoker	Re	--	--
Experimenter	2.84	2.42-3.35	<0.001
Regular smoker	6.27	5.10-7.75	<0.001
**Membership in a sport team**	1.30	1.14-1.49	<0.001
**Having physical fight**	2.58	2.25-296	<0.001
**Doing general risk taking behavior**	2.67	2.23-3.20	<0.001
**Living with parents**	1.52	1.15-2.02	0.003
**Age**	1.09	1.07-1.11	<0.001
**Self-esteem**	0.92	0.91-0.93	<0.001

To adjust the possible cofounding effect of other variables, we performed multiple logistic regression model. The results of this analysis can be seen in **Table 3**. According to the results of this table, after adjustment for other factors, cigarette smoking as an experimenter (OR=1.94,95%CI:1.61-2.34, P<0.001)) and regular (OR=3.80,95%CI:2.96-4.89, P<0.001) compared to nonsmokers, age (OR=1.08,95%CI:1.03-1.12, P<0.001), membership in a sport team (OR = 1.26,95%CI:1.08-1.48, P=0.004), having physical fight (OR = 2.18,95%CI:1.85-2.57, P<0.001), doing general risk taking behavior (OR = 2.05,95%CI: 1.66-2.53, P<0.001), and self-esteem (OR=0.93,95%CI:0.92-0.95, P<0.001) were associated with NSSI.

**Table 3:** Multiple logistic regression analysis of the association between “non-suicidal self-injury" and predictors in a sample of Iranian students.

	Non-suicidal self-injury		
	*Adjusted estimation*		
Variables	OR	95%CI	P
**Gender (being male)**	0.80	0.63-1.00	0.055
**Being university student**	0.78	0.59-1.04	0.094
Cigarette smoking status			
Non smoker	Re	--	--
Experimenter	1.94	1.61-2.34	<0.001
Regular smoker	3.80	2.96-4.89	<0.001
**Membership in a sport team**	1.26	1.08-1.48	0.004
**Having physical fight**	2.18	1.85-2.57	<0.001
**Doing general risk taking behavior**	2.05	1.66-2.53	<0.001
**Living with parents**	0.93	0.66-1.32	0.684
**Age**	1.08	1.03-1.12	<0.001
**Self-esteem**	0.93	0.92-0.95	<0.001

## Discussion

In the present study, the effect of demographic factors and high-risk behaviors on NSSI was investigated in a sample of Iranian high school and university students. In general, our study identified some modifiable and non-modifiable risk factors that were related to the experience of NSSI. The prevalence of NSSI in the present study was 24.4%. Smoking, being a member of a sports team, having physical fights, and engaging in high-risk behaviors have a direct relationship with NSSI. Additionally, having high self-esteem was a protective factor.

According to the present study, there was a 24.4% prevalence of NSSI among the participants. The study in Tabriz revealed that adolescents had a 6.2% prevalence of NSSI (4). The prevalence of NSSI in this study is lower than that of Turkish(28.5) and Indian (31.2) university students (8, 30). Marin and et al. believe that this difference may be due to the assessment tool, age of the participants and definition of NSSI in the studies conducted in different studies (4).

Our results showed that cigarette smoking (experimenter and regular smoker) and general risk-taking behavior are individual factors affecting NSSI in study participants that are consistent with previous studies (4, 31). Xiao et al. demonstrated that smoking has a positive relationship with NSSI (32). Evidence shows that cigarette smoking and high-risk behaviors cause many mental health problems and illnesses, including depression and anxiety, which peak in adolescence and early adulthood, resulting in unpleasant feelings such as self-loathing and anger, and loneliness. Thus, NSSI may be a way to eliminate these feelings and cope with problems. Also, because of the relative calm after committing these behaviors, NSSI becomes a chronic behavior in these people (33–36).

The results of our study showed that gender has no significant relationship with the occurrence of NSSI. Despite the fact that men had a higher incidence of NSSI than women. In a study in Iran, Marin et al. showed that women were more likely to experience NSSI, but there was no significant difference between the prevalence and incidence of self-injury and gender in their study (4). Wang et al. found that adolescent girls were more susceptible to NSSI behaviors than their male counterparts (17). In a meta-analysis by Bresin et al., which included a relatively large number of studies with diverse and non-biased demographic samples, there was strong evidence that women were more likely than men to engage in NSSI. They demonstrated that NSSI can vary among adolescent boys and girls. In addition, they believe that hormonal differences between women and men (e.g., testosterone vs. estradiol) affect gender differences in NSSI occurrence (37). On the other hand, most of the self-injuring behaviors in women compared to men can be attributed to the occurrence of more mental disorders such as depression and anxiety in this group, so that the rate of depression in women is 3 times higher than men (38, 39).

The present study showed that membership in a sport team increases the odds of NSSI. Boone et al. showed that increasing sports motivation is associated with a decrease in the frequency of NSSI (40). The findings of Southerland et al. also suggested that membership in sports teams is associated with a reduced risk of thoughts, plans, and self-harm, so examining this indicator helps screen for behaviors that lead to self-injury (41). There is limited information in the field of social activities and NSSI, so more comprehensive study in this field is recommended.

Previous studies have shown that people who behave aggressively towards others are also more likely to harm themselves (42, 43). These results are in line with the present study. Also, Myklestad and et al. showed that depression, anxiety and parental conflict can explain the relationship between bullying and self-harm. These factors show the importance of the family environment and emotional regulation (44). NSSI and violence against others both seem to be rooted in a major problem for mental disorders, so screening for aggression and harm to others should also be screened when assessing people who have hurt themselves.

Our findings showed that self-esteem is a protective factor for NSSI, so that a higher score of self-esteem reduces the odds of NSSI. In line with the present study, Nagy and et al. believe that low self-esteem is associated with increased motivation to self-punish, thus leading to more intense involvement in NSSI in individuals (45). In a meta-analysis study, people who involved in NSSI reported lower self-esteem (46). The results of various studies showed that low self-esteem and self-confidence in adolescents and young people are associated with NSSI (47, 48). Low self-esteem is one of the main symptoms of emotional and psychological disorders that can be accompanied by many painful feelings, such as despair, guilt, and sadness. People resort to NSSI to get rid of these tensions and punish themselves because they have a sense of inferiority, disgust, and hatred towards themselves, causing NSSI and relief from these painful feelings (19).

Baetens et al. Showed that family dysfunction, lack of emotional relationships, and excessive behavioral control and monitoring lead to behavioral problems in children and, ultimately, NSSI (49). Another study showed that adolescents who live with both parents are less likely to harm themselves due to better quality of life, security, behavioral problems by parents, and improved health status (13). The present study also showed that living with parents acts as a protective factor against self-harm in the multivariate model, although this relationship is not significant.

## Strengths and limitations

There are several advantages to this study. First. This study is conducted on Iranian high school and university students in a large population. Second, the response rate was high. Additionally, there were several limitations to this study. The cross-sectional design of the current study prevents the determination of causal relationships. In addition, we did not include many possible (diagnosed and/or treated) and important psychiatric disorders (including personality disorder) in this study due to lack of data on these disorders.

## Conclusion

In the present study we evaluated the prevalence of NSSI and its determinants among high school and university students. We also evaluated the impact of some risk aking behaviors and other related factors on NSSI. This study showed the prevalence of NSSI was relatively high among high school and university students.This study revealed that regular cigarette smoking had the highest OR among all variables and only self-esteem was a protective factor for experience of NSSI. More research is required to identify the root causes of NSSI, as well as its diagnosis, prevention, and treatment for teenagers and young adults in Iran.

## Data Availability

The raw data supporting the conclusions of this article will be made available by the authors, without undue reservation.
